# An integrated model for rice supplier selection strategies and a comparative analysis of fuzzy multicriteria decision-making approaches based on the fuzzy entropy weight method for evaluating rice suppliers

**DOI:** 10.1371/journal.pone.0301930

**Published:** 2024-04-18

**Authors:** Ghazi M. Magableh

**Affiliations:** Industrial Engineering Department, Faculty of Hijjawi for Engineering Technology, Yarmouk University, Irbid, Jordan; Istinye University: Istinye Universitesi, TURKEY

## Abstract

Rice, being a staple food in many countries, necessitates the identification of reliable suppliers to ensure a steady supply. Consequently, it is vital to establish trustworthy vendors for various types of this essential grain who can meet stringent product quality standards. This study aims to identify, analyze, rank, and select primary rice suppliers. The study emphasizes the importance of selecting and managing suitable providers to meet customer demands, proposes a ranking model for rice suppliers, and introduces developed fuzzy MCDM techniques. It proposes an integrated model for selecting rice suppliers, considering factors related to the processes before, during, and after selecting providers within a defined framework. The outcomes shows that rice supplier selection strategy can efficiently identify reliable rice suppliers, improve buyer value, reduce procurement risk, enhance efficiency, and establish strong supply chain relationships in complex decision-making processes. To assess suppliers, the study introduces two advanced integrated approaches and compares them. The fuzzy entropy weight method (EWM) was used to determine the criteria weights. The ranking of rice suppliers was achieved using a fuzzy multi-objective optimization based on ratio analysis (MOORA), fuzzy complex proportional assessment (COPRAS), and combinations of these two methods in different approaches. The methodology supports decision-makers in a rapidly evolving global environment by assisting importers, traders, suppliers, procurement, and logistics management, particularly for non-rice-cultivating countries in rice importation and supplier selection. The numerical analysis is grounded in a real-world case study of selecting rice suppliers in Jordan. The findings reveal that the various strategies yield both similar and different results. Furthermore, the integrated method is considered the most accurate for evaluating rice imports and suppliers, aligning closely with the reality of the current situation.

## 1. Introduction

Rice is a fundamental grain in the diets of people worldwide, with a significant portion of the global population heavily dependent on it as a food source. Consequently, the demand for premium rice continues to grow. This demand has led to the emergence of numerous rice suppliers worldwide, each offering a unique array of products and services. The supplier selection process (SSP) is a complex, multi-criteria task that considers both qualitative and quantitative factors. The SSP aids in structuring supplier bases and improving the overall efficiency of the supply chain. The SSP aims to reduce purchasing risk, enhance the total value for the buyer, and establish strong, enduring relationships between buyers and suppliers.

Rice is the second largest grain food product imported by Jordan, following wheat, and it also ranks second in terms of consumption. Unlike wheat, which is imported, stored, distributed, and strategically controlled by the government [1], rice is entirely imported by private traders, albeit under government supervision. The private sector handles the importation, storage, sale, and distribution of rice, adhering to the terms and standards established by the Jordanian government to ensure quality and maintain strategic reserves. Jordan imports various types of rice, including long, medium, short, and brown grains, with medium-grain rice accounting for most consumption.

Although Jordan is a small nation that does not produce rice, its average annual per capita consumption is approximately 27 kilograms. Jordanians consume between 400 and 500 tons of rice daily. Medium-grain rice, because of its stickier texture and suitability for regional dishes, such as Mansaf, is favored by Jordanian consumers. Over the past few years, rice from the United States has dominated the market because of its superior quality and cooking properties [2, 3]. The United States Department of Agriculture reports that rice is a fundamental part of the Jordanian diet, with the medium-grain variety, Camolino, being the most popular. The Jordan–US Free Trade Agreement, enacted in 2010, abolished import taxes between the two countries, making the importation of high-quality American rice economically viable [4]. The Jordanian market presents a broad spectrum of options from various suppliers, each with distinct characteristics [5]. Jordan imports all its rice, primarily from the United States, Italy, Thailand, India, Pakistan, Portugal, Russia, and Egypt [6, 7].

To the best of our knowledge, there is little research on the rice supply chain in Jordan, including a notable lack of logistical studies addressing the supply, storage, and distribution of local rice products. Prior research has not addressed the classification of top-tier rice suppliers in the region, particularly considering evolving global conditions, such as water scarcity, climate change, worldwide crises, shifting consumption patterns, and the annual increase in demand. Consequently, this study aims to fill this research gap by selecting and ranking the most suitable suppliers of rice grain to Jordan, given the current environmental and statistical conditions. The unique nature of rice as a strategic import, a staple in daily family meals, is particularly importance considering that Jordan imports all the rice it consumes. The primary contribution of this research is the development of a comprehensive model and strategy for selecting rice suppliers, focusing on procedures before, during, and after the selection process. It includes the following key essential elements: supplier selection phases, criteria, optimization, challenges, and monitoring and management. The study presents various approaches for evaluating suppliers, including fuzzy EWM, FMOORA, FCOPRAS, and integrated FMOORA-FCOPRAS methods. It also presents hybrid fuzzy multicriteria decision-making (MCDM) approaches for ambiguous data.

The goal is to introduce a strategy and effective methodology for selecting and ranking rice suppliers under different conditions, present novel fuzzy MCDM methods to rank rice suppliers, and emphasize the importance of selecting and managing suitable providers to meet customers’ changing demand. This study aims to address the following key questions: What factors influence rice importation? How are the best suppliers determined? Who are the primary rice suppliers? How can the most reliable suppliers be identified? What criteria are essential for selecting rice suppliers? How do various fuzzy MCDM methods differ in selecting the optimal supplier? The primary objective of this study is to develop an innovative model for a rice supplier selection strategy (RSSS) and identify and rank the best rice suppliers given the current global and local conditions. The study also sets out to achieve several other goals, such as identifying best practices for supplier identification in fluctuating circumstances, establishing criteria for supplier comparison, selecting trustworthy suppliers, and strategizing for future supplier selection amidst changes. This research aids in the selection of rice suppliers, considering various external variables and local stakeholders, including government agencies, private sectors, associations, trade unions, consumer protection organizations, wholesalers, retailers, and consumers. This study serves as a foundation for examining logistic systems and supply chains for rice in the region and assists in the future selection and ranking of reliable suppliers. Additionally, it introduces and compares various fuzzy MCDM methods.

**Fig 1:**
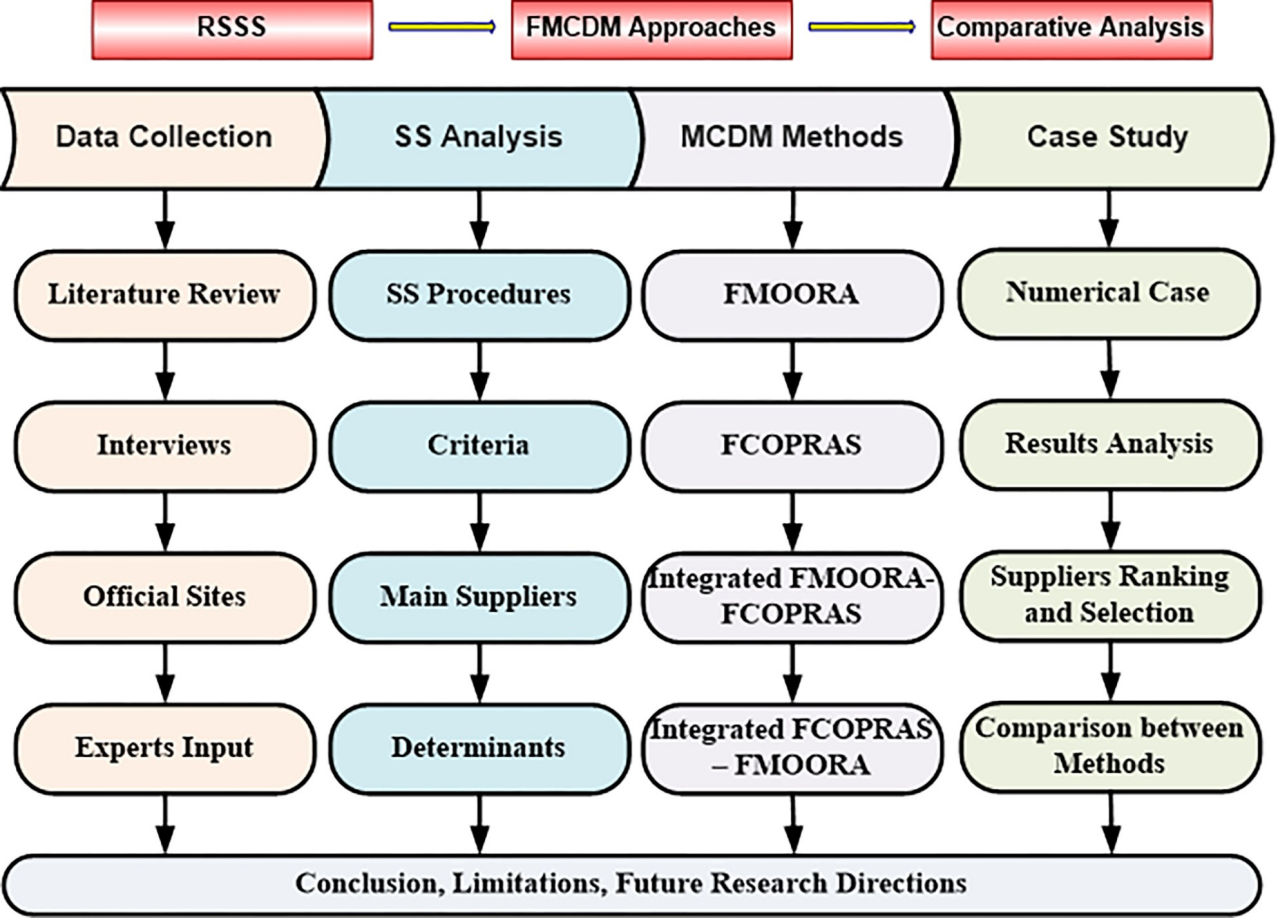
Research methodology.

As shown in Fig 1, the study is divided into four primary phases. The first phase involves the data and information collection through a review of prior studies, interviews with stakeholders from both the public and private sectors, and rice importers. This phase also includes the examination of official websites of relevant government agencies and contributions of a team of experts specializing in rice and supply chains. The second phase identifies optimal practices for supplier selection, determining the key criteria, identifying the primary rice suppliers to Jordan, and recognizing the challenges and barriers in rice importation. The third phase presents the MCDM methods used in this study, along with an explanation of the steps in each method. The fourth and final phase applies the theory to a numerical case study to rank and select suppliers using each method and evaluates and compares the different methods. The research obtained approval from the Scientific Research Ethics Council at Yarmouk University with a reference number IRB/2023/451. An informed consent form to participate in the research was sent to each participant and participant written consent was obtained in advance. The questionnaire was distributed and approvals and answers to the questionnaire questions were obtained during the period from 23 October 2023 to 8 December 2023.

The remainder of this paper is organized as follows. Section 2 provides a review of relevant prior research. Section 3 introduces the rice supplier selection strategy (RSSS). Section 4 introduces the fuzzy MCDM methods, describing the steps involved and mathematical formulation of each method. Section 5 offers a numerical analysis of a case study, aiming to rank and select the most suitable suppliers. Finally, Section 6 summarizes the findings, discusses the limitations, identifies the target audience, and suggests areas for future research.

## 2. Literature review

### 2.1 Agri-food SC

Recent public health issues have heightened focus on agricultural product supply chains. Therefore, agri-food supply chains (AFSCs) are facing stricter regulations and more frequent audits, suggesting that traditional supply chain processes may be subject to revision and modification [8]. The increasing structural complexity of AFSCs heightens their susceptibility to a range of risks. Effective and appropriate risk management in AFSCs is essential for enhancing performance [9]. Using advanced technologies can contribute to the sustainability of agri-food production [10]. Supply chain management procedures, including information exchange, supplier relationships, and logistical integration, can significantly and positively influence AFSC performance [11]. Supplier selection is a vital component in establishing a competitive supply chain. For an AFSC to be sustainable, it is essential to develop a sustainable supplier selection decision model, leading to improved responsiveness through enhanced business performance, reliable suppliers, and strong supplier relationships [12].

### 2.2 Rice supplies

The AFSCs of various grains, such as wheat, rice, and maize, include numerous operations, ranging from planting and harvesting to distribution. AFSC management aims to ensure an adequate supply and integration of operations to reduce costs. Rice, a staple in global diets, is an important agricultural commodity [13]. It has unique supply chain characteristics rooted in its high demand, high price, product diversity, and extensive production and consumption areas [14]. The rice supply chain is marked by discrete data, complicated connections, and several hazardous materials. Enhancing the information management and control capabilities of the rice supply chain is a crucial strategy to ensure rice quality and safety [15] and address numerous issues in rice trading [16]. A consistent supply of rice is vital for food security. The numerous participants in the rice supply chain have complex economic and socioenvironmental relationships, complicating the implementation of sustainability [17].

### 2.3 Supplier selection

Supplier selection, a critical process in identifying the optimal agri-food provider becomes particularly challenging given the multitude of suppliers and unstable economic conditions [18]. Thus, evaluating the rice supply chain and refining supplier selection is an essential step in rice supply chain management [19]. Indeed, the selection of providers is a pivotal aspect of supply chain management. The task of selecting a supplier is difficult, even though accurate predictions of unknown variables can potentially mitigate the negative effects on supplier selection [20]. The challenge of selecting the best rice provider often breeds competition. Moreover, selecting an efficient supplier can yield benefits such as reduced production costs, improved product quality, timely delivery, and the flexibility to meet customer requirements. The complexity of supplier selection is evident because decision-makers must understand both qualitative and quantitative aspects of the rice supply chain [21]. Decisions regarding the evaluation, selection, and performance management of suppliers carry significant weight for businesses because of the high purchase cost-to-revenue ratio [22]. Resilience planning is increasingly becoming a strategic imperative in the SSP to maintain service provision in a highly competitive global environment [23]. However, only a handful of studies have addressed supplier selection issues in AFSCs using hybrid methods of economics and supplier selection criteria [18].

### 2.4 MOORA and COPRAS methods

Vendor selection is a critical task for achieving business success and competitiveness because it often involves unique organizational requirements. Therefore, it is necessary to review whether potential suppliers meet these requirements. Various methods have been proposed for this purpose. For instance, the fuzzy analytic hierarchy process (AHP) and MOORA techniques have been used to evaluate and select vendors [24]. The fuzzy MOORA (FMOORA) approach has been employed to assess the potential for mushroom farming [25], and MOORA has been used to select the best teachers and staff [26]. Fuzzy MOORA has also been applied to select resources for mushroom growth based on a new scoring system [27]. The probabilistic FCOPRAS technique has been used to address uncertainty issues and select a biodegradable dynamic plastic product [28]. The complex proportional assessment (COPRAS) supplier selection approach has been examined for its applicability and capability [29], and the COPRAS method has been used for multiple attribute group decision-making in a picture fuzzy environment to select a green supplier [30]. The multi-criteria COPRAS method based on parametric measures for intuitive fuzzy sets has been used to select green suppliers [31]. Finally, a hybrid MCDM model of the fuzzy picture-induced preference relations and their consistency analysis (PIPRECIA) and fuzzy COPRAS approaches have been suggested for selecting truck tractors [32].

Supplier selection has always posed a fundamental challenge for any manufacturing organization. Choosing the optimal supplier from a multitude of options is a complex task for managers because it involves various types of evaluation interactions and rules. Several MCDM techniques have been employed to address this issue. For instance, the additive ratio assessment (ARAS), COPRAS, and MOORA methods, integrated with fuzzy AHP, have been used for supplier selection [33]. The MOORA and COPRAS techniques have been applied to select sustainable suppliers [34] and evaluate and rank green vendors [35]. The combination of MOORA, grey relational analysis (GRA), and COPRAS techniques has been used in an analytic network process to select green suppliers [36]. The simple additive weighting (SAW), MOORA, and COPRAS techniques have been used to identify potential mining locations [37]. The MOORA and COPRAS techniques have been compared for supplier evaluation, chosen for their simpler calculation steps and minor differences in average final ranking values [38]. Lastly, fuzzy COPRAS, fuzzy MOORA, and fuzzy multi-objective optimization by ratio analysis plus the full multiplicative form (MULTIMOORA) techniques have been combined for sustainable supplier evaluation and selection of green supply chains within reverse logistics [39].

### 2.5 SC in Jordan

Establishing robust, enduring relationships with suppliers enables Jordanian firms to optimally meet their requirements in terms of timing and location, thereby giving them a competitive edge in fulfilling customer demands and enhancing their responsiveness [40]. Jordanian businesses should focus on cultivating strategic supplier partnerships, selecting suppliers based on quality, facilitating bidirectional communication for addressing complaints and suggesting improvements, and involving suppliers in planning and development processes [41]. In a highly competitive environment, supplier integration significantly and positively affects the efficiency of Jordan’s supply chain [42]. Jordanian small and medium-sized enterprises (SMEs) can greatly benefit from the use of the internet for supply chain management to improve their business management and performance [43]. Collaboration among supply chain members and effective information exchange can bridge the gap between customer expectations and supplier reality [44]. The decision of Jordanian companies to adopt green supply chain management is largely influenced by factors such as suppliers, the environment, customers, and costs [44]. The influence of information technology on supply chain performance, including firm logistics, vendor relationships, and customer relationship management, is recognized, and can alleviate challenges in Jordanian supply chain performance [45].

An examination of prior research reveals a notable lack of studies pertaining to rice in both Jordan and the Middle East and North Africa (MENA) region. Specifically, there is an absence of research concerning the supply chain and the importation, storage, and distribution of rice in Jordan. Furthermore, to the best of our knowledge, no studies have been examined the selection of rice suppliers in Jordan. This study aims to address these research gaps by proposing a strategy for selecting rice suppliers, analyzing key suppliers, ranking these suppliers based on the criteria employed, and identifying the optimal rice suppliers for Jordan. Additionally, this study introduces novel fuzzy MCDM methods for identifying and comparing suppliers.

## 3. Rice Supplier Selection Strategy (RSSS)

**Fig 2:**
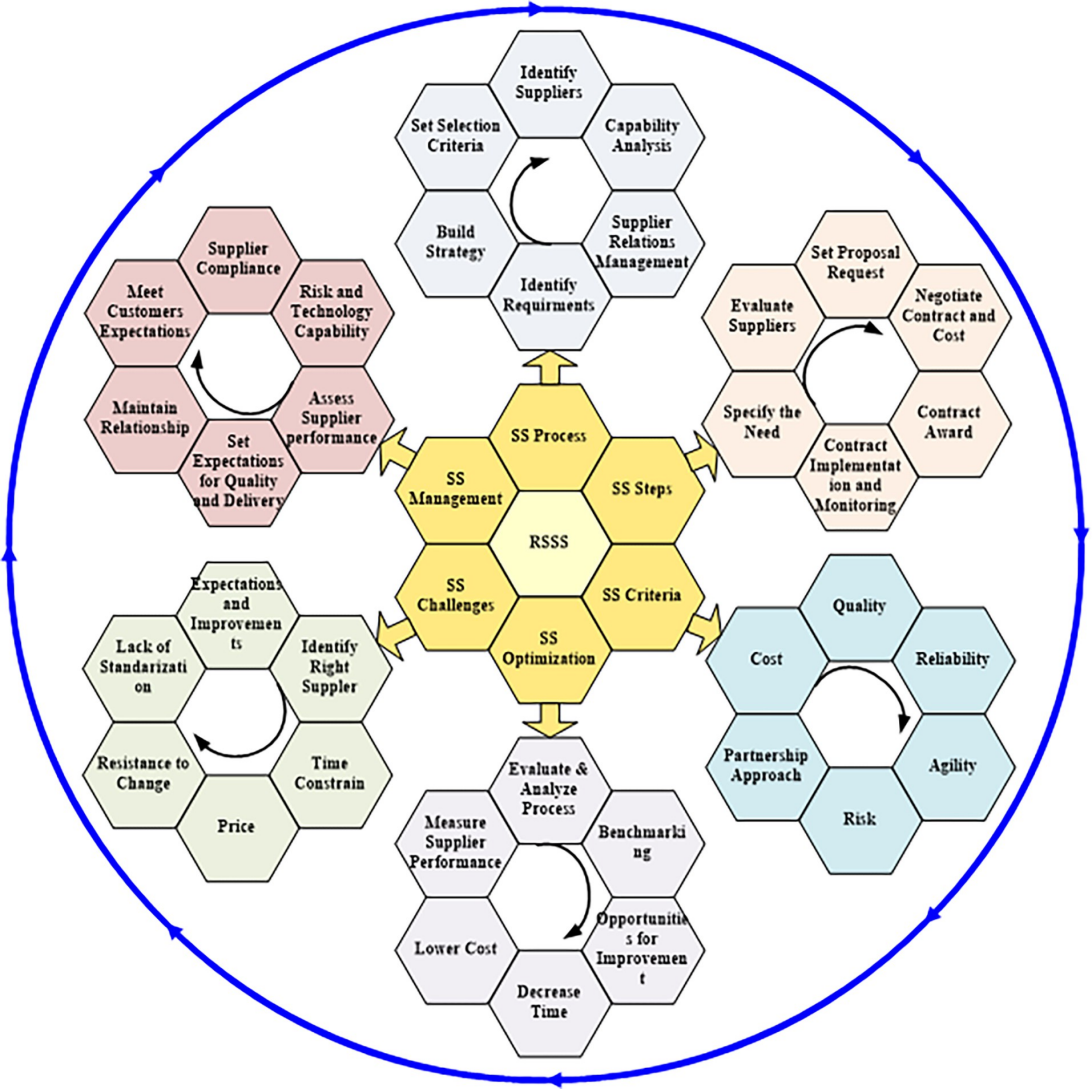
Rice supplier selection strategy framework.

Strategic sourcing and the precise identification of alternative suppliers are vital components of supply chain planning, and the importance of supplier selection strategies in mitigating uncertainty has been emphasized. It is crucial to choose and manage appropriate providers using the right strategy that can support the flexibility needed to meet customer demand. A significant task influencing a company’s long-term success is the creation of a sustainable, criteria-based SSP. Supplier selection is a multi-criteria problem that considers both qualitative and quantitative factors. Fig 2 presents the framework for the rice supplier selection strategy, which comprises the following six primary elements: supplier selection procedures, steps in supplier selection, supplier selection criteria, optimization of supplier selection, challenges in supplier selection, and supplier-monitoring and management. Strategic planning influences supplier performance evaluation and the criteria for supplier selection. Strategic sourcing is one of the fastest-growing management disciplines, with strategies for supplier selection central to corporate activity across various aspects of logistics. As the complexity of technology has increased, supply chains have become more complex and dynamic. Thus, to stay competitive and adapt to rapidly changing markets, it is necessary to enhance flexibility.

### 3.1 SSP

The SSP primarily aims to minimize purchasing risk, enhance the total value for the buyer, and establish robust, enduring relationships between buyers and suppliers. The SSP should strategically enable a business to identify the optimal source. This involves understanding the business’s needs, client expectations, and any legislative or industry standards the company must comply with. Developing a supplier selection strategy extends beyond merely comparing prices to choose the right supplier. The decision incorporates various factors, such as cost, quality, reliability, and service. The prioritization of these factors is guided by the business’s objectives and strategy. Therefore, the supplier selection criteria comprises a set of priorities and considerations used in selecting a vendor or supplier.

To streamline the process of narrowing down potential suppliers, it is beneficial to create a list that explicitly states the expectations and requirements of the business from a supplier. This approach can simplify the decision-making process, making it more objective, and aid in distinguishing between various vendors. The selection of a supplier is influenced by numerous unique factors, such as price, quality, service, delivery, reputation, location, reliability, and values, among others. Once the selection criteria are established, a list of potential suppliers can be compiled. This list may include current and former suppliers, competitors, business groups, previous contacts, and online sources. The ability of suppliers to meet the needs of a purchasing company includes quality requirements, timely delivery, corrective actions, and adherence to environmental and safety standards. Evaluations of supplier capabilities can help set realistic expectations for each supplier and identify their contribution to the company’s quality objectives. These assessments also provide a reliable quantitative analysis of the risks associated with a particular supplier. Supplier relationship management encourages the cultivation of long-term, mutually advantageous relationships with suppliers. It allows for the free exchange of ideas and feedback and encourages collaboration by identifying the best suppliers. Supplier segmentation, operational patterns, the right strategy, implementation, monitoring, and collaboration can all contribute to effective supplier relationship management.

### 3.2 Supplier selection steps

The process of supplier selection involves identifying, evaluating, and entering into contracts with vendors. The SSP constitutes a substantial portion of a company’s financial resources and is crucial to the success of any business. As shown in Fig 2, this framework suggests six steps for selecting the most suitable supplier. A company’s sourcing strategy should incorporate a mechanism for identifying business needs as a component of supplier selection. These needs must be prioritized in this step because this enables the company to find, evaluate, and choose the most appropriate supplier to meet these needs.

The first step in the supplier assessment process involves evaluating the business’s requirements and generating a list of specifications to identify suitable suppliers for engagement or discussion. Suppliers are subsequently assessed based on a set of selection criteria, which includes determining how to assign a score to each supplier based on these factors. Key elements of the evaluation process include the evaluation purpose, questions and criteria, schedule and work plan, data collection tools and methodologies, analysis, and recommendations for action. Suppliers are requested to submit a proposal outlining their approach to addressing the specified issue in response to a pre-established request for proposals (RFP). Drafting an RFP is a crucial step in this process. This request is reviewed by potential bidders, who then suggest enhancements. The final RFP is issued after considering these suggestions. Subsequently, bidders submit their proposals. The contract negotiation process aims to reach a mutually beneficial agreement to secure favorable terms and minimize operational, legal, and financial risks. Supplier negotiations amalgamate interpersonal skills, strategies, market knowledge, and data-driven insights.

Negotiating prices with suppliers is essential because it can lead to cost savings and improved deals. A key aspect of procurement involves selecting a competent vendor or bidder, which ensures that the most qualified business secures the contract. This process of assessing proposals and assigning the appropriate contract is referred to as contract awarding. By this stage, the suitability of the proposals is typically determined. A written contract implementation outlines the services that the company will receive from the outsourced supplier. It comprises a set of best practices and guidelines that aid companies in ensuring their contracts are adhered to. The process of ensuring parties comply with a contract is termed contract monitoring. This also involves evaluating and tracking the performance and status of the contract to ensure that the obligations outlined in the contract are being fulfilled as planned.

### 3.3 Supplier selection criteria

A supplier selection criterion refers to a set of priorities used to select suppliers and vendors. This decision is influenced by various unique attributes, known as selection criteria, which include cost, quality, service, reliability, timing, and reputation. The business must determine how much it is willing to invest in supplies, considering the reliability and quality of the providers. Striking the right balance between cost, reliability, quality, and service is crucial. Thus, conducting a supplier cost analysis is vital for evaluating current suppliers and determine whether they offer the best value for their products. The quality of the suppliers’ products must remain consistent to prevent customers from associating them with low quality. Moreover, if a supplier disappoints a customer by delaying delivery or providing defective goods, it could lead to customer dissatisfaction. Therefore, it is essential to compare the quality of products from high-end and less expensive suppliers. The reliability of suppliers directly affects the dependability and credibility of a company’s products. Suppliers are tasked with ensuring the uninterrupted flow of goods, components, and services necessary for business operations. It is crucial to verify that a supplier has a stable cash flow, enabling them to fulfill orders punctually. Conducting a credit check can provide assurance that they will not cease operations when their services are most needed. Supplier agility allows a business to quickly respond to unforeseen emergencies or shifts in customer demand. Speed, flexibility, and responsiveness are essential characteristics of agile suppliers. To meet the ever-changing demands of the supply chain, a company must possess the agility to promptly alter its strategy, particularly in the areas of delivery, inventory management, and procurement.

Supplier risk can include various issues, such as operational and organizational challenges, supplier delays, disruptions in manufacturing, natural disasters, theft, shortages, and cybersecurity threats. The process of supplier risk management involves the identification, assessment, and management of potential risks associated with supplier interactions. A supplier partnership represents a long-term commitment to mutual collaboration, aimed at benefiting both parties involved. This partnership can be facilitated by sharing relevant information and balancing the risks and rewards inherent in the relationship, thereby enabling both parties to achieve their objectives effectively. A strategic partnership comprises several components, including vision and strategy, values, investment, planning and management systems, communication, and the balance between risk and reward. Such partnerships can also decrease issues related to availability, delays, and quality.

### 3.4 Supplier selection optimization

Supplier optimization refers to the process of assessing and improving a supplier’s performance to meet an organization’s requirements. This process aims to establish a more efficient and effective supply chain capable of delivering superior quality goods and services at reduced costs. Continuous evaluation and monitoring of the SSP are crucial to create a robust and reliable network that aligns with the company’s goals. Factors such as the supplier’s financial stability, product quality, delivery speed, and customer service should all be considered.

Supplier analysis involves assessing supplier performance to identify areas requiring improvement. However, supplier benchmarking is the process of comparing a supplier’s performance against its leading competitor or the top-performing company in its industry. This benchmarking process facilitates the comparison of vendors against a set of established standards for both ongoing and completed contracts. Benchmarking can be conducted in terms of process, performance, and strategy. The business can then adopt these strategies to achieve comparable or superior results. Supplier improvement is a critical business strategy that a company can employ. It augments the performance and capabilities of suppliers, providing the company with a competitive advantage.

Tactics for managing suppliers can vary from simple evaluations to more intricate strategies, such as technology transfer and innovation. A key step in meeting customer demand promptly and reducing inventory costs is reducing supplier lead times. To expedite supplier selection, it is necessary to choose a method, implement it, and use software to assess, select, and negotiate supply contracts with suppliers. A company can decrease its supplier-related costs by implementing a supplier cost reduction strategy, which could involve negotiations, altering the procurement process, or identifying new suppliers. By collaborating with fewer but more efficient suppliers, companies can reduce their expenditure on purchased goods and services. Supplier cost optimization includes all supply chain activities and flows. There are various methods to assess supplier performance, including evaluating measures of quality, innovation, delivery, cost, compliance, and responsiveness. Each company assigns different levels and weights to these performance measures based on its strategy and requirements. By consistently assessing supplier performance, companies can compare suppliers, identify their strengths and weaknesses, and set targets and incentives for improvement. The main steps in measuring supplier performance include establishing metrics, identifying potential suppliers, developing an evaluation process, understanding business needs, setting performance indicators, strengthening supplier relationships, and identifying risks.

### 3.5 Supplier selection challenges

The process of supplier selection presents numerous challenges, making it difficult for management to identify the optimal supplier amidst a myriad of global, regional, and local changes, production fluctuations, currency exchange rate fluctuations, and global crises. These challenges include identifying the right supplier, time constraints, cost considerations, resistance to change, lack of standardization, and the need for experience and continuous improvement. Selecting the appropriate supplier can be complex because it requires evaluating the supplier’s skills, knowledge, and compatibility with the company’s needs. The time-intensive nature of this process poses a significant barrier because companies may not have sufficient time to thoroughly assess each potential supplier. Consequently, time constraints can lead to rushed decisions and suboptimal supplier selection, which can adversely affect the company. The pressure to meet deadlines may necessitate rushed decisions, potentially resulting in an inadequate evaluation of suppliers.

Cost is a crucial factor in supplier selection because of its influence on current and future budgets. When selecting a supplier, organizations must balance the cost of the product or service against its perceived value and benefits. To be chosen, the supplier must offer competitive prices, while demonstrating their ability to deliver high-quality products, thereby providing the best value for money. Organizations may face resistance from employees accustomed to a specific supplier, making change difficult. This resistance can delay supplier selection or, worse, lead to the selection of an inappropriate solution. The lack of standardization in the vendor selection process can result in variations in selection criteria and evaluation procedures, potentially leading to inaccurate results. Therefore, organizations must implement a consistent methodology to ensure a fair and unbiased evaluation. Vendors often respond to RFPs using their own templates, complicating comparisons. If business and technical requirements are not clear and explicit, the SSP can become quite challenging. It is essential for businesses to clearly outline requirements to ensure the product or service is delivered as expected. Without clear requirements, miscommunications between the business and supplier are inevitable, disrupting the work environment and adding unnecessary stress. These requirements often include the scope of work, budget, performance objectives, and other relevant information. This clarity is crucial for vendors to effectively present their solution and demonstrate how it best meets the client’s needs [46].

### 3.6 Supplier selection management

Supplier management is a structured program designed to manage suppliers and increase their influence on the purchasing company. This program involves tracking supplier deliverables, collaborating to develop new processes, ensuring compliance, and managing invoice payments. Suppliers’ performance is evaluated and monitored through a process known as supplier assessment, which can be conducted periodically, such as quarterly or annually. This assessment should examine the supplier’s financial health, the quality of their products or services, turnaround times, and customer service. The expectations for a supplier include ethical conduct and compliance with environmental, safety, and health standards; avoidance of money laundering, conflicts of interest, anti-competitive behavior, and bribery and corruption; and adherence to international trade regulations. Supplier quality refers to a supplier’s ability to meet customer requirements with their products. The delivery schedule is a mutual agreement between the buyer and supplier regarding the timing and frequency of product deliveries. It is a plan that details the specifics of future delivery periods, which can be mutually agreed upon or set by the buyer. By maintaining a long-term beneficial relationship, the supplier and organization can exchange ideas and feedback, enhancing operations, streamlining the supply chain, reducing costs, and improving customer service.

The SSP aims to identify appropriate suppliers who can fulfill an organization’s objectives and requirements and obligations and the expectations of its customers. To maintain quality at the source, the supplier must consistently meet or exceed current and future customer expectations and needs. Supplier compliance aims to ensure that suppliers adhere to a company’s specific standards, needs, legal requirements, and ethical guidelines during procurement. Supplier risk refers to the potential for financial losses or disruptions in business operations when dealing with suppliers. This risk can stem from various issues, such as supplier insolvency, substandard quality, delayed deliveries, or ethical breaches. Technological risks include those that could lead to the purchased product or service being incomplete, underperforming, or malfunctioning. Digital technology allows suppliers to operate with efficiency and structure, with all activities monitored in a single location. This technology simplifies the process for buyers to find and evaluate supplier proposals, aiding suppliers in finding new customers and receiving feedback on their products. Technology provides benefits by reducing costs, improving customer service, and enhancing operational efficiency. In today’s business environment, companies must have access to advanced procurement technology because it streamlines the process and allows all stakeholders to gain real-time visibility and identify opportunities for cost savings.

## 4. Supplier evaluation methods

This study proposes a novel hybrid MCDM method that combines fuzzy COPRAS (FCOPRAS) and fuzzy MOORA (FMOORA) methods. This is an integrated approach to address decision-making challenges under fuzzy information conditions, specifically in selecting the most suitable alternative from a group of options. Two innovative integrated methods are proposed for supplier evaluation, including a comparative analysis of these methods. The fuzzy entropy weight method (EWM), that is, F-EWM, is used to determine the criteria weights. The FMOORA, FCOPRAS, integrated FMOORA-FCOPRAS, and integrated FCOPRAS-FMOORA methods are used to rank rice suppliers.

### 4.1 Apply EWM to calculate criteria weights

The entropy method uses the response value to assign a weight to each criterion, considering both inter-criterion and intra-criterion significance. These weights are then applied to the FMOORA, FCOPRAS, and hybrid FMOORA-FCOPRAS MCDM methods. The process of determining the weights of the criteria using the entropy method is as follows [47, 48]:

*Step 1*: Normalize the decision matrix

The standardized value of the *i*th sample in the *j*th criterion is represented as *r*_*ij*_, and is calculated using Eq (1):

$$r_{ij}=\frac{X_{ij}}{\sum_{i=1}^{m}X_{ij}}\;i=1,2,\ldots ,n;\;j=1,2,\ldots ,m,$$ (1)

where *m* is the number of experts or samples, *n* is the number of criteria, and the measured value of the *i*th expert in the *j*th criterion is denoted as x_ij_.

*Step 2*: Calculate the entropy of each criterion

The EWM entropy value *e*_*j*_of the *j*th criterion is defined in Eqs 2 and 3:

$$e_{j}=-k\sum_{i=1}^{m}r_{ij}\;\ln\;r_{ij}\;i=1,2,\ldots ,m;\;j=1,2,\ldots ,n$$ (2)

wherek=1ln(m). (3)

*Step 3*: Calculate the weight vector for the criteria

The range of the entropy value *e*_*j*_ is [0, 1]. However, because the differentiation degree of index *j* increases with *e*_*j*_, the index should be given more weight. Consequently, the weight *w*_*j*_ is calculated as follows:

$$W_{j}=\frac{1-e_{j}}{\sum_{j=1}^{n}(1-e_{j})}\;j=1,2,\ldots ,n.$$ (4)

This study introduces a new approach that uses fuzzy sets to conduct a fuzzy evaluation using triangular fuzzy numbers (TFN). Subsequently, it extracts the weights for the criteria using the EWM.

### 4.2 The FMOORA method

The MOORA is a compensatory method, typically used for quantitative attributes. It is particularly effective in simultaneously ranking both desirable and undesirable criteria. This method has been extensively applied in various selection processes, including supplier optimization, contractor selection, material selection, sustainable logistics, site selection, and risk analysis. A key advantage of the MOORA method is its computational efficiency, requiring a minimal number of calculations. The fundamental principle of the MOORA method is to evaluate the overall performance of each alternative by calculating the difference between the sums of its normalized performance related to cost and benefit criteria. This study slightly modifies the steps to implement the fuzzy MOORA approach [25, 49–53].

### 4.3 The FCOPRAS method

The COPRAS method offers an optimal solution corresponding to an appropriate answer, facilitating comparisons of options in terms of superiority. The primary advantage of the COPRAS method over other MCDM methods is its ability to determine the degree of utility, showing which option is better or worse than other alternatives. FCOPRAS is employed to aid decision-makers in selecting the most effective strategy in a fuzzy environment, where ambiguity and uncertainty are controlled by linguistic concepts parameterized by TFNs. This method helps evaluate the maximum and minimum index values, considering their individual effects on the results assessment. One application of the COPRAS method is in supplier selection across various fields and disciplines. This study proposes a fuzzy COPRAS method for supplier evaluation that can be applied to the SSP logically and efficiently. As with MOORA, we modify the FCOPRAS steps to demonstrate how the mathematical concept of FCOPRAS operates [48, 54–59].

This study uses the F-EWM, FCOPRAS, and FMOORA techniques and the newly developed integrated FMOORA-FCOPRAS and FCOPRAS-FMOORA methods. These are used as fuzzy MCDM techniques to evaluate the weight of each criterion and importance of alternatives. These methods are derived from the FMOORA and FCOPRAS approaches [25, 48–61]. The integrated methods are discussed in detail in the subsequent sections.

### 4.4 The integrated FMOORA-FCOPRAS method

*Step 1*: Identify the decision problem. Form the committee of decision-makers and experts (*E*_1_, *E*_2_,…,*E*_*k*_), identify the goal, select criteria, and define the feasible alternatives. Assume there are *K* experts (*E*_*k*_, *t* = 1,2,…,*K*) responsible for evaluating *m* options (*A*_*i*_, *i* = 1,2,…,*m*) considering the weight of each criterion *n* (*C*_*j*_, *j* = 1,2,…,*n*).

*Step 2*: Select the appropriate linguistic variables. Each necessary linguistic variables should be represented by a positive TFN. These linguistic variables should be used to calculate the significant weights of criteria and conduct evaluations of alternatives based on multiple criteria. Before defining these linguistic variables as positive TFN, it is important to first identify the linguistic variables for the significant weights of the criteria and fuzzy scores for the alternatives for each criterion.

*Step 3*: Establish the fuzzy significance weights for the evaluation criteria. The criterion weight is determined using the EWM, as shown in Eqs (1)–(4). The fuzzy significance weight ${\tilde{w}}_{i}$ of criterion *C*_*j*_ is given as follows:

$${\tilde{w}}_{j}=\frac{1}{k}({\tilde{w}}_{j}^{1}⊕{\tilde{w}}_{j}^{2}⊕\ldots ⊕{\tilde{w}}_{j}^{k}),$$ (5)

where $w_{j}^{k}$ is the fuzzy weight of criterion *C*_*j*_, as specified by the *k*th assessor, and ${\tilde{w}}_{j}^{k}=(l_{j}^{k}⊕m_{j}^{k}⊕u_{j}^{k})$.

*Step 4*: After the decision-makers have evaluated the options and criteria, the following Eq (6) is used to sum the evaluations of the alternatives and criteria to create an aggregated fuzzy decision matrix:

$${\tilde{x}}_{ij}=\frac{1}{k}({\tilde{x}}_{ij}^{1}⊕{\tilde{x}}_{ij}^{2}⊕\ldots ⊕{\tilde{x}}_{ij}^{k}),$$ (6)

where $x_{ij}^{k}$ is the evaluation rating for alternative *A*_*i*_ under criterion *C*_*j*_ evaluated by the *k*th decision-maker; ${\tilde{x}}_{ij}^{k}=(l_{ij}^{k}⊕m_{ij}^{k}⊕u_{ij}^{k})$, where $l_{ij}^{k},m_{ij}^{k},u_{ij}^{k}$ represent the lower, middle, and upper values of TFNij, respectively.

*Step 5*: Build the fuzzy decision matrix $\tilde{D}$ and criteria weight vector $\tilde{W}$. Create a fuzzy decision matrix using input from experts, measuring each criterion using a triangle membership function. TFNs are usually used to rate alternatives related to decision criteria by translating linguistic characteristics into fuzzy rates. The value of the aggregated weights is expressed in matrix format for the supplier selection problem. The ratings of *m* options in relation to *n* criteria are then used to create a decision matrix, $\tilde{D}$, as follows:

$$\tilde{D}=\left[\begin{array}{cccc} {\tilde{x}}_{11} & {\tilde{x}}_{12} & \ldots & {\tilde{x}}_{1n}\\ {\tilde{x}}_{21} & {\tilde{x}}_{22} & \ldots & {\tilde{x}}_{2n}\\ \vdots & \vdots & \ddots & \vdots \\ {\tilde{x}}_{m1} & {\tilde{x}}_{m2} & \ldots & {\tilde{x}}_{mn} \end{array}\right],\;i=1,2,\ldots ,m;\;j=1,2,\ldots ,n,$$ (7)

where *x*_*ij*_ is a linguistic variable denoted by the TFN score of alternative *A*_*i*_ (*i* = 1,2) related to the attribute *C*_*j*_ (j = 1,2,…,*n*).

The fuzzy weight vector is given by Eq (8), which is as follows:

$$\tilde{W}=({\tilde{w}}_{1},{\tilde{w}}_{2},\ldots ,{\tilde{w}}_{n}).$$ (8)

*Step 6*: Calculate the normalized fuzzy decision matrix $\tilde{Y}$. In this step, the fuzzy decision matrix is normalized using the following Eq (9):

$$\tilde{Y}={\left[{\tilde{y}}_{ij}\right]}_{mxn},$$ (9)

where ${\tilde{y}}_{ij}=(y_{ij}^{l},y_{ij}^{m},y_{ij}^{u})$ are calculated as follows:

$$y_{ij}^{l}=\frac{x_{ij}^{l}}{\sqrt{\sum_{i=1}^{m}\left[{\left(x_{ij}^{l}\right)}^{2}+{\left(x_{ij}^{m}\right)}^{2}+{\left(x_{ij}^{u}\right)}^{2}\right]}}$$ (10)

$$y_{ij}^{m}=\frac{x_{ij}^{m}}{\sqrt{\sum_{i=1}^{m}\left[{\left(x_{ij}^{l}\right)}^{2}+{\left(x_{ij}^{m}\right)}^{2}+{\left(x_{ij}^{u}\right)}^{2}\right]}}$$ (11)

$$y_{ij}^{u}=\frac{x_{ij}^{u}}{\sqrt{\sum_{i=1}^{m}\left[{\left(x_{ij}^{l}\right)}^{2}+{\left(x_{ij}^{m}\right)}^{2}+{\left(x_{ij}^{u}\right)}^{2}\right]}}$$ (12)

*Step 7*: Calculate the weighted normalized decision matrix $\tilde{F}$. The weighted normalized decision matrix $\tilde{F}$ is given by the following Eq (13):

$$\tilde{F}={\left[{\tilde{f}}_{ij}\right]}_{mxn},$$ (13)

where ${\tilde{f}}_{ij}={\tilde{y}}_{ij}(.){\tilde{w}}_{j}$ and ${\tilde{f}}_{ij}=(f_{ij}^{l},f_{ij}^{m},f_{ij}^{u})$.

*Step 8*: Calculate the normalized assessments of the alternatives. Calculate the overall benefit ratings. Eqs (14)–(16) calculate the overall ratings of a substitute for the beneficial criteria for lower, medium, and upper values, respectively:

$$z_{i}^{+l}=\sum_{j=1}^{n}f_{ij}^{l}|j\in J^{max}$$ (14)

$$z_{i}^{+m}=\sum_{j=1}^{n}f_{ij}^{m}|j\in J^{max}$$ (15)

$$z_{i}^{+u}=\sum_{j=1}^{n}f_{ij}^{u}|j\in J^{max}.$$ (16)

Eqs (17)–(19) calculate the overall ratings of a substitute for the nonbeneficial criteria for lower, medium, and upper values, respectively:

$$z_{i}^{-l}=\sum_{j=1}^{n}f_{ij}^{l}|j\in J^{min}$$ (17)

$$z_{i}^{-m}=\sum_{j=1}^{n}f_{ij}^{m}|j\in J^{min}$$ (18)

$$z_{i}^{-u}=\sum_{j=1}^{n}f_{ij}^{u}|j\in J^{min}.$$ (19)

*Step 9*: Calculate the relative importance of each option using Eq (20). Here, *Q*_*i*_ represents the proportional weight:

$$Q_{i}=Z_{i}^{+}+\frac{\underset{i}{\min}z_{i}^{-}\sum_{i=1}^{m}z_{i}^{-}}{z_{i}^{-}\sum_{i=1}^{m}\frac{\underset{i}{\min}z_{i}^{-}}{z_{i}^{-}}},$$ (20)

where the minimum values of *Z*_*min*_ = min *Z*_*i*_, *for i* = 1,2,…,*m*.

*Step 10*: Defuzzify each factor’s fuzzy weight and fuzzydecision matrix using Eq (21), which is as follows:

Zi(zi−,zi+)=[(zi+l−zi−l)+(zi+m−zi−m)+(zi+u−zi−u)]3 (21)

*Step 11*: Determine the priority or importance of the alternatives. Establish the alternatives order of importance based on their respective weights. The alternative with the highest relative weight is the most preferable option, and the alternative with the highest relative weight has greater priority (rank). The optimality criterion *A* is determined using Eq (22). The higher the weight (rank) *Q*_*i*_ of the alternative, the larger is its weight. The highest level of satisfaction is *Q*_*max*_, given as follows:

$$A^{*}=\{A_{i}|\underset{i}{\max}Q_{i}\}$$ (22)

*Step 12*: Determine the degree of utility for all the following alternatives *N*_*i*_ (Eq (23)), where, Q_i_ and Q_max_ are the weights of the alternatives from Eq (20):

$$N_{i}=\left(\frac{Q_{i}}{Q_{max}}\right)\times 100\%$$ (23)

### 4.5 The integrated FCOPRAS-FMOORA method

Steps 1–5 are the same as those for the FMOORA-FCOPRAS method.

*Step 6*: Calculate normalized fuzzy decision matrix $\tilde{Y}$. In this step, the fuzzy decision matrix is normalized. The normalized values are determined using the following Eq (24):

$$\tilde{Y}={\left[{\tilde{y}}_{ij}\right]}_{mxn},$$ (24)

where ${\tilde{y}}_{ij}=(y_{ij}^{l},y_{ij}^{m},y_{ij}^{u})$ are calculated as follows:

$$y_{ij}^{l}=\frac{x_{ij}^{l}}{\sum_{i=1}^{m}x_{ij}^{l}}$$ (25)

$$y_{ij}^{m}=\frac{x_{ij}^{m}}{\sum_{i=1}^{m}x_{ij}^{m}}$$ (26)

$$y_{ij}^{u}=\frac{x_{ij}^{u}}{\sum_{i=1}^{m}x_{ij}^{u}}.$$ (27)

*Step 7*: The weighted normalized decision matrix $\tilde{F}$ is calculated as in Step 7 of the FMOORA-FCOPRAS method.

*Step 8*: Calculate the beneficial and nonbeneficial criteria $z_{i}^{-},z_{i}^{+}$ as in Step 8 of the FMOORA-FCOPRAS method.

*Step 9*: Calculate the defuzzified overall index *Z*_*i*_. This stage determines the overall performance index *Z*_*i*_ for each choice. The vertex approach is used to generate the defuzzified values of the overall ratings for the beneficial and nonbeneficial criteria for each alternative, given as follows:

$$Z_{i}(z_{i}^{-},z_{i}^{+})=\sqrt{\frac{1}{3}[{\left(z_{i}^{+l}-z_{i}^{-l}\right)}^{2}+{\left(z_{i}^{+m}-z_{i}^{-m}\right)}^{2}+{\left(z_{i}^{+u}-z_{i}^{-u}\right)}^{2}]}$$ (28)

*Step 10*: Rank the alternatives based on their overall performance indices. The option with the highest index is the most advantageous option.

## 5. Case study

This study’s quantitative analysis is grounded in a real-world case of rice supplier selection in Jordan. The primary objective is to rank and choose the most suitable rice suppliers for the current import situation, based on the established criteria. Jordan, under government supervision, imports rice from over 10 countries via the private sector. Traders have the liberty to import rice from their preferred sources and determine the quality, specifications, and quantity to import. These decisions are influenced by factors such as the competition level among merchants, local market consumption volume, and consumer preferences for specific sources and types of rice. The government’s role is to oversee the quality and quantities of imported rice to safeguard public health and maintain a strategic reserve for a period determined by the state’s strategic plans. Medium-grain rice from the United States is the most consumed and preferred type of rice in Jordan, fitting well with the popular meals and daily dishes prepared in homes, restaurants, and public feasts.

Rice is currently imported by traders from various countries across multiple continents. However, the primary six countries that export the most rice to Jordan via local traders were chosen for this study. To maintain the confidentiality of information and protect the competitive nature and business operations of private sector traders, the names of these countries remain undisclosed. Each importing trader has a unique trademark used for local rice sales and marketing, which, for this study, has been replaced with Supplier 1, Supplier 2, and so on, up to Supplier 6 (S1, S2, S3, S4, S5, S6). To conduct preliminary evaluations and determine alternatives and criteria, a team of experts is assembled. This team comprises five academics, traders, relevant officials, and specialists in supply chain and logistics systems.

Numerous criteria are identified for evaluating key providers, including cost, quality, reliability, lead time, agility, risk, exchange rate, distance, taxes, and partnership approach. However, a panel of experts and traders identified the following five primary criteria: cost (C), distance (D), quality (Q), reliability (R), and taxes (T). The cost criterion refers to the expense of purchasing rice from the exporting source or country. Distance is measured in kilometers and represents the nautical distance between the Gulf of Aqaba, Jordan’s only seaport and the only exporting port in the country. This measurement excludes internal transportation, storage, and distribution operations because these are considered part of the total supply chain costs and vary according to the importer. Quality refers to the rice specifications based on previously discussed standards, government-set standards, and international standards that vary by type and source. Reliability assesses the dependability, steadiness, trustworthiness, and credibility of the suppliers, a crucial factor for establishing long-term, mutually beneficial partnerships with key suppliers. Taxes refer to the fees imposed by the government on imports. This factor is significant because Jordan has free trade agreements with certain countries that do not impose taxes or fees on imported goods. The quality and reliability factors are evaluated by experts, while the remaining factors are assessed based on currently available real data.

### 5.1 The integrated FMOORA-FCOPRAS method

The experts assessed the significance of criteria C1, C2,…, C5 and rated each supplier (S1, S2,…, S6) based on the perspectives of the decision-makers and in accordance with the criteria. Table 1 presents the linguistic variables used to assign the correct weight to each criterion and the expert assessment for each supplier related to the applied criteria. The table also shows the corresponding linguistic values of the TFN used to evaluate the criteria and options.

**Table 1:** Linguistic terms and the corresponding TFN.

Linguistic variable	TFN	
	Criteria weight	Alternative rating
Very Low (VL)	(0,0.1,0.2)	(0,1,2)
Low (L)	(0.1,0.2,0.3)	(1,2,3)
Medium Low (ML)	(0.2,0.3,0.4)	(2,3,4)
Medium (M)	(0.4,0.5,0.6)	(4,5,6)
Medium High (MH)	(0.5,0.6,0.7)	(5,6,7)
High (H)	(0.6,0.7,0.8)	(6,7,8)
Very High (VH)	(0.8,0.9,1)	(8,9,10)

The importance of each criterion is determined using the F-EWM method (Eqs (1)–(5)). Table 2 presents the fuzzy weights of the criteria, derived from expert evaluations, and the defuzzified values of *e j* and *w j*. These fuzzy normalized weights are used in subsequent calculations. The experts use the linguistic variables to assess the importance of each criterion.

**Table 2:** Fuzzy criteria weights using the F-EWM method.

Expert/ Criteria	C	D	Q	R	T
**E1**	M	MH	VH	VH	VH
	(0.4,0.5,0.6)	(0.5,0.6,0.7)	(0.8,0.9,1)	(0.8,0.9,1)	(0.8,0.9,1)
**E2**	M	H	H	M	H
	(0.4,0.5,0.6)	(0.6,0.7,0.8)	(0.6,0.7,0.8)	(0.4,0.5,0.6)	(0.6,0.7,0.8)
**E3**	VH	H	H	VH	H
	(0.8,0.9,1)	(0.6,0.7,0.8)	(0.6,0.7,0.8)	(0.8,0.9,1)	(0.6,0.7,0.8)
**E4**	H	M	M	VH	ML
	(0.6,0.7,0.8)	(0.4,0.5,0.6)	(0.4,0.5,0.6)	(0.8,0.9,1)	(0.2,0.3,0.4)
**E5**	M	H	H	M	H
	(0.4,0.5,0.6)	(0.6,0.7,0.8)	(0.6,0.7,0.8)	(0.4,0.5,0.6)	(0.6,0.7,0.8)
**Fuzzy Entropy**	(0.47,0.529,0.583)	(0.488,0.545,0.597)	(0.518,0.573,0.597)	(0.534,0.588,0.637)	(0.484,0.543,0.597)
**Normalized Fuzzy weight**	(0.212,0.212,0.213)	(0.204,0.205,0.205)	(0.192,0.192,0.205)	(0.186,0.185,0.185)	(0.206,0.206,0.205)
**ej**	0.527	0.543	0.563	0.587	0.541
**wj**	0.211	0.204	0.195	0.185	0.205

The real values associated with the criteria of cost, distance, and taxes are documented, and an expert assessment is employed for the criteria of quality and reliability. This expert assessment uses a scale from 1 to 7, where 1 indicates a low rating and 7 signifies high importance. Table 3 presents the evaluation of the rice suppliers, along with the corresponding linguistic terms and values. Additionally, the fuzzification process is executed based on the weighted set of criteria to avoid bias in the selection and specification of these criteria. The outcomes of the fuzzification are shown in Table 3.

**Table 3:** Fuzzification of the equivalent criteria values for evaluating suppliers.

Linguistics scale			Criteria				
Linguistic term	Initialization	TFN	C (JODs)	D (Km)	Q	R	T %
Very Low	VL	(0,1,2)	<0.25	<100	1	1	0
Low	L	(1,2,3)	0.25–<0.40	100–<500	2	2	0–<0.1
Medium Low	ML	(2,3,4)	0.40–<0.5	500–<1000	3	3	0.1–<0.2
Medium	M	(4,5,6)	0.50–<0.70	1000–<3000	4	4	0.2–<0.3
Medium High	MH	(5,6,7)	0.70–<0.90	3000–<5000	5	5	0.3–<0.5
High	H	(6,7,8)	0.90–<1.0	5000–<10000	6	6	0.5–<0.7
Very High	VH	(8,9,10)	≥1.0	≥10000	7	7	≥0.7

Table 4 presents the consolidated fuzzy decision matrix. The assessments from the experts are aggregated using Eq (6). The fuzzy linguistic evaluations of suppliers in relation to the criteria, along with the associated TFN, are computed using Eq (8).

**Table 4:** Aggregated fuzzy decision matrix.

Suppler/ Criteria	C	D	Q	R	T
**S1**	M	VH	VH	H	VL
	(4,5,6)	(8,9,10)	(8,9,10)	(6,7,8)	(0,1,2)
**S2**	ML	ML	H	VH	H
	(2,3,4)	(2,3,4)	(6,7,8)	(8,9,10)	(4,5,6)
**S3**	M	MH	MH	MH	M
	(4,5,6)	(5,6,7)	(5,6,7)	(5,6,7)	(4,5,6)
**S4**	M	MH	M	MH	M
	(4,5,6)	(5,6,7)	(4,5,6)	(5,6,7)	(4,5,6)
**S5**	L	H	H	MH	H
	(1,2,3)	(6,7,8)	(6,7,8)	(5,6,7)	(4,5,6)
**S6**	ML	H	H	MH	M
	(4,5,6)	(5,6,7)	(4,5,6)	(5,6,7)	(4,5,6)

The normalized fuzzy decision matrix is shown in Table 5 and based on Eqs (9)–(12).

**Table 5:** Normalized fuzzy decision matrix.

	C	D	Q	R	T
**S1**	(0.53,0.508,0.492)	(0.58,0.558,0.541)	(0.548,0.529,0.515)	(0.424,0.423,0.422)	(0,0.089,0.147)
**S2**	(0.265,0.305,0.328)	(0.145,0.186,0.216)	(0.411,0.412,0.412)	(0.566,0.544,0.527)	(0.447,0.445,0.442)
**S3**	(0.53,0.508,0.492)	(0.363,0.372,0.379)	(0.343,0.353,0.361)	(0.354,0.362,0.369)	(0.447,0.445,0.442)
**S4**	(0.53,0.508,0.492)	(0.363,0.372,0.379)	(0.274,0.294,0.309)	(0.354,0.362,0.369)	(0.447,0.445,0.442)
**S5**	(0.132,0.203,0.246)	(0.435,0.434,0.433)	(0.411,0.412,0.412)	(0.354,0.362,0.369)	(0.447,0.445,0.442)
**S6**	(0.265,0.305,0.328)	(0.435,0.434,0.433)	(0.411,0.412,0.412)	(0.354,0.362,0.369)	(0.447,0.445,0.442)

Next, generate the weighted normalized fuzzy decision matrix using Eq (13). The results are presented in Table 6.

**Table 6:** Weighted normalized fuzzy decision matrix.

	C	D	Q	R	T
**S1**	(0.249,0.269,0.287)	(0.283,0.304,0.323)	(0.284,0.303,0.321)	(0.227,0.249,0.269)	(0,0.048,0.088)
**S2**	(0.124,0.161,0.191)	(0.071,0.101,0.129)	(0.213,0.236,0.257)	(0.302,0.32,0.336)	(0.217,0.242,0.264)
**S3**	(0.249,0.269,0.287)	(0.177,0.203,0.226)	(0.178,0.202,0.225)	(0.189,0.213,0.235)	(0.217,0.242,0.264)
**S4**	(0.249,0.269,0.287)	(0.177,0.203,0.226)	(0.142,0.169,0.193)	(0.189,0.213,0.235)	(0.217,0.242,0.264)
**S5**	(0.062,0.107,0.143)	(0.212,0.237,0.258)	(0.213,0.236,0.257)	(0.189,0.213,0.235)	(0.217,0.242,0.264)
**S6**	(0.124,0.161,0.191)	(0.212,0.237,0.258)	(0.213,0.236,0.257)	(0.189,0.213,0.235)	(0.217,0.242,0.264)

Next, each alternative’s overall benefit and cost ratings are calculated. Table 7 shows the fuzzy performance values (Z) in terms of the benefit (advantages) and non-benefit (disadvantages, costs, or loss) criteria. The TFNs of the benefit and non-benefit criteria are individually computed. Here, *Z*^+^ represents the beneficial values and *Z*^−^ represents the nonbeneficial values.

**Table 7:** Normalized fuzzy performance values.

	*Z*^+^ (B)	*Z*^−^ (NB)
**S1**	(0.511,0.552,0.59)	(0.532,0.621,0.698)
**S2**	(0.515,0.556,0.593)	(0.412,0.505,0.584)
**S3**	(0.366,0.416,0.46)	(0.642,0.713,0.777)
**S4**	(0.331,0.382,0.428)	(0.642,0.713,0.777)
**S5**	(0.402,0.449,0.492)	(0.491,0.586,0.666)
**S6**	(0.402,0.449,0.492)	(0.553,0.64,0.713)

Next, Eq (20) is used to calculate the overall ratings for the beneficial and non-beneficial criteria for the rice suppliers. Table 8 shows the relative fuzzy importance of each option.

**Table 8:** Relative fuzzy weight *Q*_*i*_ of each option.

	*Q*_*i*_		
Alts	*l*	*m*	*u*
**S1**	0.885919	0.552212853	0.589878155
**S2**	0.805703	0.555855326	0.592858943
**S3**	0.819481	0.415525567	0.459953316
**S4**	0.783974	0.381809055	0.427844705
**S5**	0.748269	0.44924208	0.492061927
**S6**	0.792159	0.44924208	0.492061927

To establish the priority of the options, calculate the defuzzified *Q*_*i*_ values. Table 9 shows the crisp values of each option’s priority or importance *Q*_*i*_ using Eqs (21) and (22) and the degree of utility for all alternatives *N*_*i*_ using Eq (23). The compared options are prioritized based on their respective weights. Table 9 shows the defuzzified *Q*_*i*_ values, utility degree *N*_*i*_, and the rank of alternatives from the FMOORA-FCOPRAS method.

**Table 9:** Overall performance index values and rank assignment of all components for the rice supplier selection.

Alts	Qi	Ni	Rank
**S1**	0.676003	1	1
**S2**	0.651472	0.96371202	2
**S3**	0.564987	0.83577517	4
**S4**	0.531209	0.78580902	6
**S5**	0.563191	0.83311879	5
**S6**	0.577821	0.85476071	3

The order of suppliers, from best to worst, is as follows: S1 > S2 > S6 > S3 > S5 > S4.

### 5.2 The integrated FCOPRAS-FMOORA method

The aggregated fuzzy decision matrix is calculated using the same steps 1–5 as in the integrated FMOORA-FCOPRAS method shown in Tables 1–4. Table 10 shows the normalized fuzzy decision matrix calculated from Eqs (24)–(27).

**Table 10:** Normalized fuzzy decision matrix.

	C	D	Q	R	T
**S1**	(0.235,0.217,0.207)	(0.25,0.237,0.227)	(0.229,0.22,0.213)	(0.176,0.175,0.174)	(0,0.038,0.063)
**S2**	(0.118,0.13,0.138)	(0.063,0.079,0.091)	(0.171,0.171,0.17)	(0.235,0.225,0.217)	(0.2,0.192,0.188)
**S3**	(0.235,0.217,0.207)	(0.156,0.158,0.159)	(0.143,0.146,0.149)	(0.147,0.15,0.152)	(0.2,0.192,0.188)
**S4**	(0.235,0.217,0.207)	(0.156,0.158,0.159)	(0.114,0.122,0.128)	(0.147,0.15,0.152)	(0.2,0.192,0.188)
**S5**	(0.059,0.087,0.103)	(0.188,0.184,0.182)	(0.171,0.171,0.17)	(0.147,0.15,0.152)	(0.2,0.192,0.188)
**S6**	(0.118,0.13,0.138)	(0.188,0.184,0.182)	(0.171,0.171,0.17)	(0.147,0.15,0.152)	(0.2,0.192,0.188)

Table 11 shows the fuzzy weighted normalized decision matrix calculated using Eq (13).

**Table 11:** Fuzzy weighted normalized decision matrix.

	C	D	Q	R	T
**S1**	(0.05,0.046,0.044)	(0.051,0.049,0.047)	(0.044,0.042,0.041)	(0.033,0.032,0.032)	(0,0.008,0.013)
**S2**	(0.025,0.028,0.029)	(0.013,0.016,0.019)	(0.033,0.033,0.033)	(0.044,0.042,0.04)	(0.041,0.04,0.039)
**S3**	(0.05,0.046,0.044)	(0.032,0.032,0.033)	(0.027,0.028,0.029)	(0.027,0.028,0.028)	(0.041,0.04,0.039)
**S4**	(0.05,0.046,0.044)	(0.032,0.032,0.033)	(0.022,0.023,0.025)	(0.027,0.028,0.028)	(0.041,0.04,0.039)
**S5**	(0.012,0.018,0.022)	(0.038,0.038,0.037)	(0.033,0.033,0.033)	(0.027,0.028,0.028)	(0.041,0.04,0.039)
**S6**	(0.025,0.028,0.029)	(0.038,0.038,0.037)	(0.033,0.033,0.033)	(0.027,0.028,0.028)	(0.041,0.04,0.039)

Next, determine the classification of the benefits criteria from Eqs (14)–(16) and costs criteria from Eqs (17)–(19). Table 12 shows both the fuzzy beneficial and nonbeneficial criteria.

**Table 12:** Fuzzy beneficial and nonbeneficial values.

	*Z*^+^ (B)			*Z*^−^ (NB)		
	*l*	*m*	*u*	*l*	*m*	*u*
**S1**	0.076736	0.074619505	0.072968525	0.10090483	0.102519	0.103471
**S2**	0.076681	0.07451453	0.072834503	0.07884403	0.083365	0.086507
**S3**	0.054792	0.055925263	0.056701178	0.12290647	0.117975	0.115161
**S4**	0.049299	0.051238581	0.052617588	0.12290647	0.117975	0.115161
**S5**	0.060285	0.060611946	0.060784767	0.09195764	0.095713	0.09784
**S6**	0.060285	0.060611946	0.060784767	0.10440391	0.104931	0.105169

Next, establish the order of the alternatives using the crisp values for the weighted normalized fuzzy decision matrix. Table 13 shows the calculated defuzzified overall index *Z*_*i*_ using Eq (28) and ranks of the alternatives.

**Table 13:** Overall assessment values.

Alts	*Z*_*i*_	Rank
**S1**	0.04363	5
**S2**	0.01634	6
**S3**	0.09388	2
**S4**	0.10085	1
**S5**	0.05422	4
**S6**	0.0677	3

The suppliers are ranked as follows, from best to worst: S4 > S3 > S6 > S5 > S1 > S2.

### 5.3 Comparison of the methods

Table 14 compares the different fuzzy MCDM methods used in this study. The four methods used are the FMOORA, FCOPRAS, integrated FMOORA-FCOPRAS, and integrated FCOPRAS-FMOORA methods.

**Table 14:** Rice supplier selection results of the four fuzzy MCDM methods.

Alts	Rank			
	FMOORA	FCOPRAS	FMOORA-FCOPRAS	FCOPRAS-FMOORA
**S1**	5	1	1	5
**S2**	6	2	2	6
**S3**	2	5	4	2
**S4**	1	6	6	1
**S5**	4	4	5	4
**S6**	3	3	3	3

**Fig 3:**
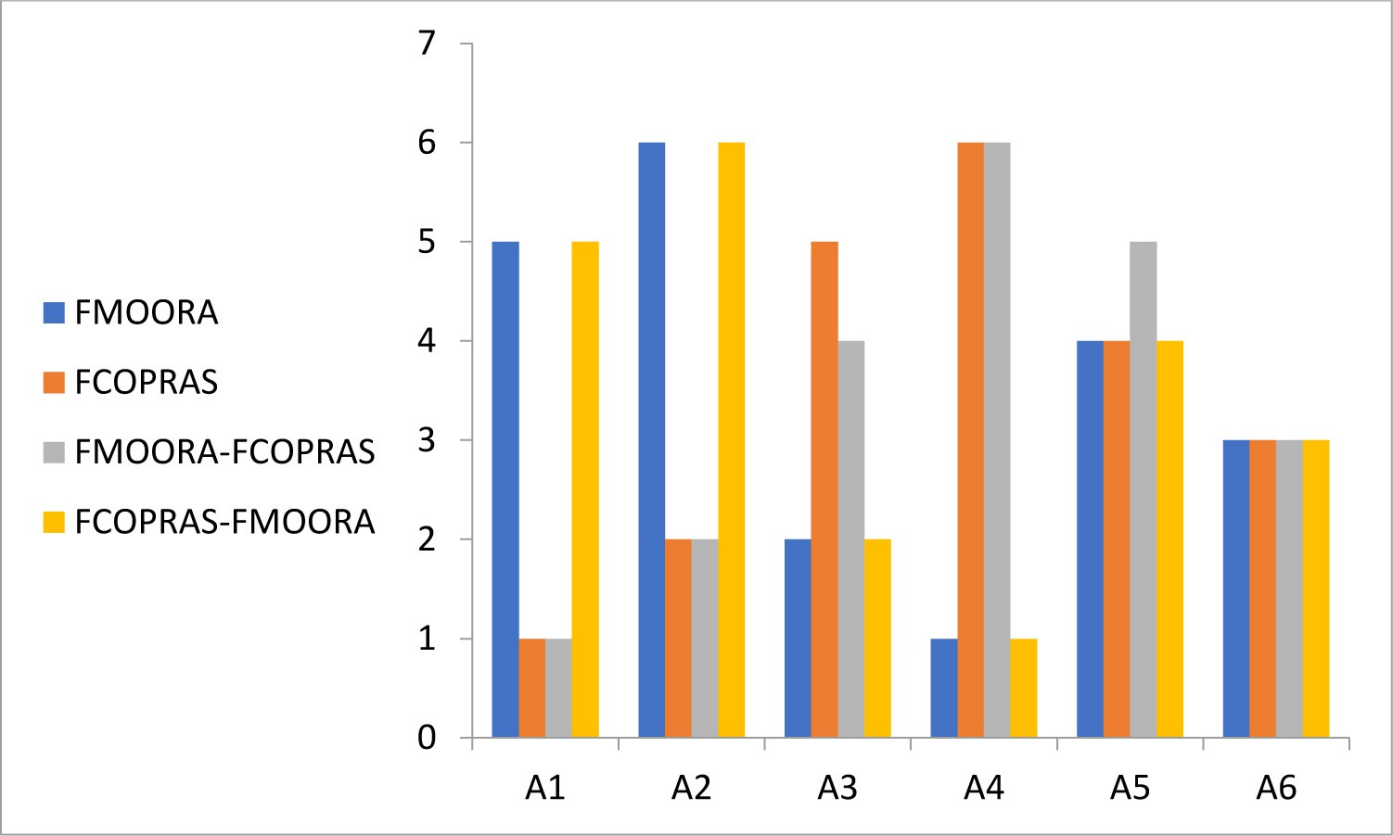
Rice supplier selection ranks based on different fuzzy MCDM methods.

Fig 3 shows the overall ranks from the rice supplier selection, showing both similarities and differences between the methods.

The comparison of the results to determine the optimal rice suppliers for the case study yields the following findings. There is a divergence in the outcomes between the FMOORA and FCOPRAS methodologies. Although the rankings of suppliers five and six (S5 and S6) are consistent across both methods, there is a variation in the rankings of suppliers S1–S4. In the FMOORA approach, the fourth supplier (S4) is ranked first, while the second supplier (S2) is ranked last. Conversely, in the FCOPRAS approach, the first supplier (S1) is ranked first and the fourth supplier (S4) is ranked last. This demonstrates a significant difference between the two methods because the supplier that ranked first in the FMOORA ranked last when using the FCOPRAS approach. The results from the FMOORA method are identical to those from the integrated FCOPRAS-FMOORA method. There is also a high degree of similarity between the results of the FCOPRAS and integrated FMOORA-FCOPRAS methods. The outcomes are largely consistent across these two methods, except for the rankings of the third and fifth suppliers.

A comparison between FMOORA-FCOPRAS and FCOPRAS-FMOORA reveals a discrepancy in the ranking results for five suppliers (S1, S2, S3, S4, S6), with a congruence observed only in the ranking of the fifth supplier (S5). In the FMOORA-FCOPRAS method, the first supplier (S1) secured the top rank, while the fourth supplier (S4) ranked sixth. Conversely, in the integrated FCOPRAS-FMOORA method, the fourth supplier (S4) achieved the top rank and the first supplier (S1) ranked fifth. The first four steps, along with the seventh and eighth steps, which involve calculating the beneficial and non-beneficial criteria and weighted normalized values, are identical across both methods. However, the methods diverge in the equations used for normalization, defuzzification, and determining the relative importance of alternatives. This divergence in methodologies accounts for the observed differences in the supplier rankings. The same pattern of similarities and differences is observed when comparing FMOORA and FCOPRAS.

The findings are shared with professionals, traders, supply chain specialists, and decision-makers to compare the outcomes and determine which approach most accurately reflects the existing conditions. They agree that the integrated FMOORA-FCOPRAS method most closely mirrors reality in terms of the current state of rice importation and supplier evaluation.

## 6. Conclusion

Rice serves as a primary food source for a significant portion of the global population, and the demand for high-quality rice continues to rise. Accordingly, there are countless rice producers worldwide, each offering a distinct array of products and services. The process of choosing a supplier is a complex decision-making task that considers how qualitative and quantitative factors interact. The SSP enhances the overall efficiency of the supply chain and facilitates the structuring of supplier bases. The SSP primarily aims to reduce procurement risk, improve the total value for the buyer, and establish robust, long-lasting relationships between buyers and suppliers. Securing reliable rice suppliers is vital to maintaining a consistent supply of rice. Therefore, it is crucial to identify dependable rice suppliers for various types of this essential crop. Rice suppliers are required to meet specific standards in terms of product characteristics and quality.

This research is the first to introduce a comprehensive strategy and model for the selection of rice suppliers, incorporating factors associated with the processes before, during, and after the selection within a defined framework. This model can serve as a valuable tool for stakeholders selecting rice suppliers, particularly in the MENA region and other non-rice producing countries. The study proposes a comprehensive model for rice supplier selection strategies, including the following six key components: phases of supplier selection, criteria for supplier selection, optimization of supplier selection, challenges in supplier selection, and monitoring and management of suppliers.

This study proposes several strategies to address challenges in decision-making when selecting the most suitable alternative. It presents two advanced integrated approaches for supplier evaluation, and includes a comparison of various methods. The weights of the criteria are determined using fuzzy EWM. The FMOORA, FCOPRAS, integrated FMOORA-FCOPRAS, and integrated FCOPRAS-FMOORA methods are used to rank rice suppliers. The hybrid fuzzy-MCDM techniques developed in this study provide a comprehensive solution to decision-making difficulties in the face of information uncertainty. The numerical analysis is grounded in a real-world example of selecting rice suppliers in Jordan. The findings reveal both similarities and differences in the outcomes produced by the different methods.

This framework is applicable for stakeholders involved in the importation and supplier selection of rice, particularly in countries that do not cultivate this crop. It is also beneficial for importers, traders, suppliers, procurement departments, and supply chain specialists in both public and private sectors. Additionally, this model is valuable for researchers, academics, and those involved in logistics management. It assists decision-makers in identifying the most suitable suppliers in an unpredictable and rapidly evolving global environment.

This research has certain limitations. These include using a specific set of criteria, a lack of comparison among all existing suppliers, local and international, and the confinement of the study to a particular global region. The scope of this study could be broadened to encompass more or all criteria to compare all global rice suppliers. Additionally, the research could be expanded to include other regions of the world. Future research could also explore other MCDM techniques to compare them and evaluate their outcomes. Lastly, future studies could incorporate additional factors and sub-criteria.
